# Automated *Mycobacterium tuberculosis* Detection in Multivariant Digitized Ziehl–Neelsen Staining Using Faster R‐CNN Method

**DOI:** 10.1155/ijbi/6692222

**Published:** 2026-01-21

**Authors:** Riries Rulaningtyas, Fashalli Giovi Bilhaq, Deby Kusumaningrum, Ronald Eric, Sayyidul Istighfar Ittaqillah, Herri Trilaksana, Dicky Bagus Widhyatmoko, Annie Anak Joseph

**Affiliations:** ^1^ Biomedical Engineering Study Program, Department of Physics, Faculty of Science and Technology, Universitas Airlangga, Surabaya, East Java, Indonesia, unair.ac.id; ^2^ Department of Medical Microbiology, Faculty of Medicine, Universitas Airlangga, Surabaya, East Java, Indonesia, unair.ac.id; ^3^ Tuberculosis Laboratory, Institute of Tropical Disease, Universitas Airlangga, Surabaya, East Java, Indonesia, unair.ac.id; ^4^ Dr. Soetomo General Hospital, Surabaya, East Java, Indonesia, jatimprov.go.id; ^5^ Biomedical Engineering Master Program, Department of Physics, Faculty of Science and Technology, Universitas Airlangga, Surabaya, East Java, Indonesia, unair.ac.id; ^6^ Department of Physics, Faculty of Science and Technology, Universitas Airlangga, Surabaya, East Java, Indonesia, unair.ac.id; ^7^ Faculty of Medicine, Universitas Airlangga, Surabaya, East Java, Indonesia, unair.ac.id; ^8^ Department of Electrical and Electronic Engineering, Faculty of Engineering, Universiti Malaysia Sarawak, Kota Samarahan, Sarawak, Malaysia, unimas.my

**Keywords:** Faster R-CNN, multivariant, sputum smear, tuberculosis, Ziehl–Neelsen

## Abstract

Tuberculosis (TB) is an infectious disease caused by *Mycobacterium tuberculosis* and remains a major public health concern in Indonesia. One of the most widely used diagnostic methods is the microscopic examination of sputum smears stained using the Ziehl–Neelsen technique. However, manual identification of TB bacteria presents significant challenges, particularly due to staining thickness variations that lead to inconsistent color intensities, making visual detection difficult and often subjective. This study is aimed at developing an automated TB bacteria detection system using deep learning, specifically the Faster R‐CNN algorithm with ResNet‐50 layers. The system is implemented using the Python programming language and the TensorFlow Object Detection API. We incorporated data augmentation in the form of random rotation, random flipping, and color processing such as hue variation, saturation stretching, brightness stretching, and exposure stretching. Experimental results show that the proposed model achieves an accuracy of 88%, with a precision of 94%, recall of 93%, and an F1‐score of 94%. The model outputs annotated images indicating the locations of TB bacteria, which can assist medical professionals in the diagnostic process. These findings demonstrate the potential of deep learning–based approaches in automating TB detection, particularly in healthcare settings with limited human resources.

## 1. Introduction

Tuberculosis (TB) is a contagious respiratory disease caused by the bacterium *Mycobacterium tuberculosis* [1, 2]. Through the release of airborne particles or droplets, TB patients can transmit this disease through the medium of the air [3]. This disease has the second‐highest mortality rate in the world after HIV/AIDS, making it a critical public health issue. The majority of TB cases occur in developing countries such as Indonesia. According to the Indonesian Ministry of Health, 397,377 TB cases were reported in 2021, although the actual number is likely much higher [4, 5]. Early detection of TB is crucial, as undetected cases may result in delayed treatment and, ultimately, death.

Automated image processing in the medical field can help in the quick and accurate diagnosis and classification of pathogens that can help save a person′s life [6]. There are several methods for detecting TB, such as a chest x‐ray test, culture test, interferon‐*γ* release assay (IGRA), GeneXpert, skin test, and microscopy test [7]. A relatively low‐cost, straightforward, safe, and effective method, often used in developing countries, is the sputum smear test [8]. This is carried out by analyzing the *M. tuberculosis* bacteria using a sputum smear image in fluorescence microscopy or conventional microscopy [9]. The method requires staining of the sputum smear sample by using the Ziehl–Neelsen stain, which can provide significant contrast to the color of the TB bacteria [10].

*M. tuberculosis* bacteria vary in length from 1 to 4 *μ*m, with a thickness ranging from 0.3 to 0.6 *μ*m. These are acid‐resistant bacteria and will turn reddish when treated with the Ziehl–Neelsen stain [11]. The level of redness given can vary, from brown, dark red, to pink [12], and is influenced by the thickness of the coloring. Variations in staining thickness make it difficult to accurately identify the bacteria and often lead to inconsistent results. Furthermore, laboratory staff still manually detect and count bacteria. This manual process requires a lot of time and effort, as they have to carefully observe a large number of images [13, 14]. A computer‐based detection system that can differentiate between images of TB bacteria, non‐TB bacteria, and background in sputum smear images can be used to solve this problem. Object detection in an image is a method to find out the possible location of an object in an image based on probability values [15]. Once the location of an object is identified, a bounding box is used to mark it and the location is then labeled. [16].

The object detection process is generally divided into region selection, feature extraction, and classification stages [17]. In the region selection stage, objects of different sizes and locations are detected within the image. A common method used to scan the entire image is the multiscale sliding window. In the feature extraction stage, the main characteristics of the objects are transformed into numerical features that represent the objects. In the classification stage, objects are visually distinguished using several classification algorithms such as support vector machines (SVMs), decision trees (DTs), and artificial neural networks (ANNs) [15, 18, 19]. An ANN is an artificial intelligence technique that consists of computational structures that can be trained to learn models from examples [20]. One of the best ANN methods used for local feature extraction is the convolutional neural network (CNN) [21].

The CNN has several different architectures, one of which is the Residual Network (ResNet), which was developed from the VGGNet architecture by adding a residual block. ResNet won several competitions in 2015, outperforming other architectures such as GoogleNet, AlexNet, and VGGNet, because of its ability to classify images on ImageNet datasets with an accuracy of 80.62% [22]. Mascarenhas and Agarwal [23] demonstrated that ResNet‐50 provides better accuracy performance compared with other CNN architectures such as VGG‐16 and VGG‐19. We use the ResNet‐50 architecture as a feature extractor because its architecture is effective at classifying small objects. We used input dimensions of 224 x 224 x 3, and the ResNet architecture performed operations on the convolution and max pooling layers using kernel of dimensions 7 x 7 and 3 x 3.

Many studies have been carried out to detect TB bacteria (including [7, 9, 22, 24–26]). Some studies ([7, 9, 24, 25]) used the CNN method with its various architectures. Studies [7, 24] used the faster region‐based convolutional neural network (Faster R‐CNN), which combines the region proposal network (RPN) with the Fast R‐CNN algorithm. These studies showed precision values of 0.826 and 0.851, recall values of 0.983 and 0.984, and F1‐scores of 0.897 and 0.912, respectively, without using any image enhancement methods. We attempted to improve the results of these parameters by using data augmentation methods in the form of random rotation, random flipping, and color processing such as hue variation, saturation stretching, brightness stretching, and exposure stretching. The Faster R‐CNN with ResNet‐50 architecture is one of the object detection methods used in deep learning, and we used it to detect the location of *M. tuberculosis* bacteria and to add a bounding box to the sputum smear image.

## 2. Materials and Methods

**Figure 2:**
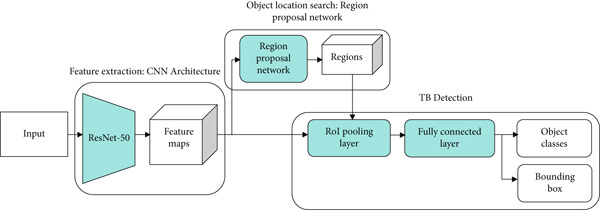
Object detection stages used in this research. The process begins with inputting the sputum smear image, followed by feature extraction using a CNN (ResNet‐50 backbone). The extracted features are then passed through a region proposal network (RPN) to localize potential bacteria regions, which are subsequently classified and refined to detect TB bacteria accurately.

The process of examining TB bacteria through the sputum smear test involves taking and preparing specimens, calculating the number of bacteria, and grouping categories based on the number of bacteria. We used 600 sputum smear images with Ziehl–Neelsen staining obtained from a published database [27]. The images were taken through a microscope with a magnification of 1000 times connected to a computer. Each image had a size of 612 x 480 pixels with three coloring qualities, namely thin, standard, and thick colors. The quality of the staining was obtained based on standard operating procedures that apply to microscopic examination of TB in Indonesia [28]. This was done so that the model had sensitivity to various coloring conditions. An example of the image can be seen in Figure 1. Figure 2 is a framework for the stages of object detection in this study.

Figure 1Sputum smear images stained using the Ziehl–Neelsen method with different levels of thickness: (a) normal thickness with clear bacteria visibility; (b) medium thickness where the background starts to interfere with visibility; and (c) excessive thickness, making bacteria harder to detect due to strong background staining [27].

**Figure:**
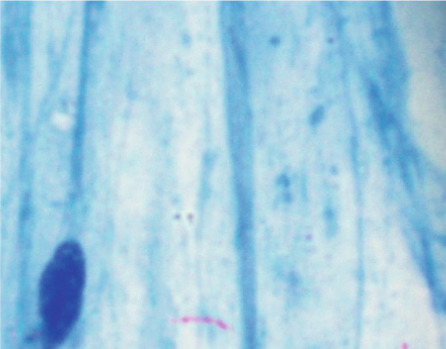
(a)

**Figure:**
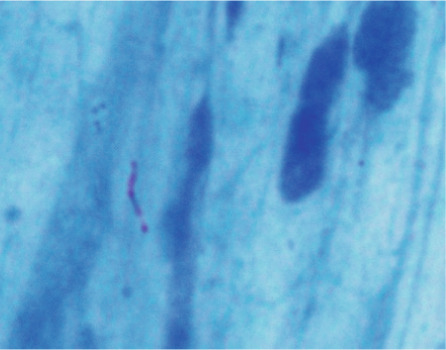
(b)

**Figure:**
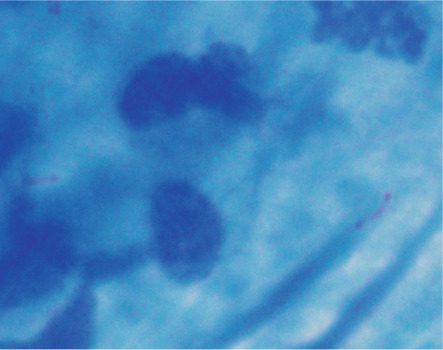
(c)

### 2.1. Data Acquisition and Data Preprocessing

First, data preprocessing was achieved by parsing the XML annotation file from all image annotations into a CSV file to be used in the training process. This can cause the resulting model to experience overfitting, where the neural network is too specific in following the training data, so that the accuracy decreases if the model is used on other inputs other than the training data [1, 22, 25, 29]. Therefore, we applied data augmentation techniques using the RoboFlow platform, generating a total of 1700 images. The augmentation process included random flipping, rotation, and several color adjustments.

The first color processing step was hue variation, where we applied hue variation by adjusting the intensity of the red, green, and blue (RGB) colors with a 3% deviation from the original image. The second step was saturation stretching, where we changed the intensity of the image′s color components evenly by a 5% deviation from the original image. The third step was brightness stretching, where we adjusted the image′s brightness level by a 10% deviation from its original value. And the fourth step was exposure stretching, where we applied exposure stretching to enhance the intensity of high‐tone colors, making the brighter regions more prominent. We also introduced a blur variation, which added data with a lower level of sharpness compared with the original image, with a variation of 0.25 pixels. From the 1700 data points, we selected 30 data points as test data (each containing 80 object classes), and the rest were used for training. Each image was annotated or given the location coordinates of the object position of each class in the image. Annotations were saved in text form using the PASCAL VOC writing standard. The annotation aims to mark the label in the form of a ground truth box as training data. There were 4100 bacterial objects from all the images that underwent the augmentation process.

### 2.2. Model Training

At this stage, the Faster R‐CNN algorithm was used, accessed from the TensorFlow Object Detection application programming interface (API) on the Google Collaboratory. TensorFlow is a platform for implementing machine learning algorithms that provides various model libraries.

#### 2.2.1. Feature Learning

**Figure 3:**
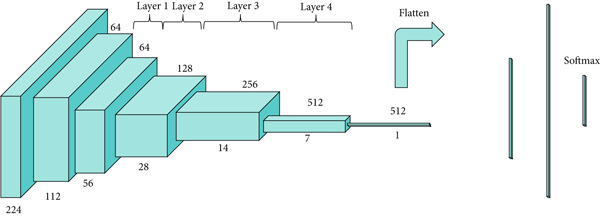
The architecture of the ResNet‐50 model used as the backbone in this research. ResNet‐50 consists of 50 layers with residual connections that help mitigate the vanishing gradient problem during training. This deep convolutional network is utilized to extract high‐level features from the input sputum smear images [22].

**Figure 4:**
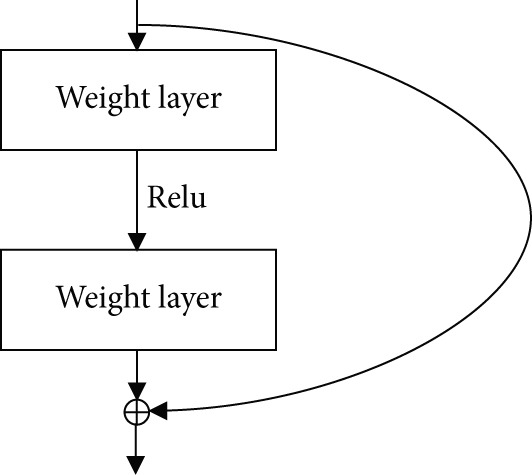
Residual layer mechanism in the ResNet architecture. This figure illustrates how input from one weight layer is passed through ReLU activation and then added to the output of a deeper layer via a shortcut connection. This residual mapping helps the network train deeper architectures by preventing vanishing gradient issues and enabling better feature learning [22].

First, feature extraction was performed using feature learning, which is the stage of model analysis on the dataset that involves a convolution process to obtain features from the related dataset [30, 31]. The backbone of the neural network itself was the basis used to perform convolution, which in this study was conducted using ResNet. The ResNet architecture is illustrated in Figure 3. In ResNet, there is an identity connection on the arrow in the form of a curve that connects the input layer with the end (output layer) of the residual block without passing through the convolutional layer, as illustrated in Figure 4. In this study, we used ResNet‐50, which contains a combination of trained model filters from the ImageNet dataset [22].

**Figure 5:**
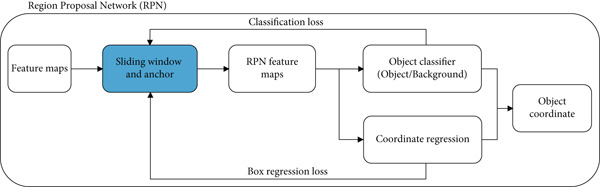
Region proposal network (RPN) used in the object detection pipeline. The RPN takes the feature maps extracted by the backbone network and generates a set of object proposals by predicting bounding box coordinates and objectness scores. These proposals are then refined and used for final classification in the detection stage.

Figure 5 shows a diagram of the RPN. RPN was achieved by placing a bounding box at the location of an image (region). A kernel moves along the last feature map of the convolution layer. One of the problems faced in deep learning is the large number of variables that need to be computed. Faster R‐CNN overcomes this problem by using anchors. In the Faster R‐CNN process, there are anchor boxes, which are a set of predefined bounding boxes as templates for placing bounding boxes and function as a scale to measure the ratio of each object in the image [32]. To be able to detect various objects in an image, Faster R‐CNN creates thousands of predefined bounding boxes, which are then selected based on the intersection over union (IoU) value of each bounding box, which indicates the accuracy of determining and placing bounding boxes in an image [33]. Usually, the principle of non‐max suppression of 0.5 is used as a threshold value of 0.5 for IoU is applied, which eliminates values less than 0.5 [34]. Faster R‐CNN is a development of Fast R‐CNN that eliminates the selective search feature, which functions to get the object location candidate, replacing it with a RPN, allowing the process of searching for proposal regions and convolution to occur in parallel.

The region of interest (RoI) pooling layer serves to reduce image dimensions, which is also known as the downsampling technique. RoI pooling retrieves a feature map from CNN after several convolutions as well as a number of object locations (proposed regions) from the RoI in the form of bounding box coordinates with great accuracy in an image. RoI is useful for generating feature maps with specific sizes, where the number of output layers will match the number of input layers. A fully connected layer connects all layers resulting from feature extraction and aims to classify data linearly by transforming the dimensions of the data held. The output of this stage will be selected again through the softmax activation layer. The softmax activation layer aims to determine whether a neuron will be reused in the next process. The softmax activation layer is an activation function that calculates the probability that each class will add up to a value of 1, and the highest probability is taken. The equation for the activation function of softmax [35] is given in Equation (1).

(1) Syi=expyi∑j=1mexpyi with j=1,⋯,m,

where  *y*_*i*_ are the input values of the softmax layer, and the output value *S*(*y*_*i*_) represents the probability that sample belongs to the *i*‐th category.

#### 2.2.2. Transfer Learning

The transfer learning stage was carried out by using a model that had been trained with a dataset as an initial but related reference for parameter adjustment with the aim of extracting features [36]. In this study, we extracted low‐level features from feature extraction, such as edge points and lines from common objects that had been trained on the ImageNet dataset. This pretrained model already had certain weights to distinguish basic features that can be trained further to recognize specific objects.

#### 2.2.3. Regularization

This stage was accomplished by calculating the loss function, which shows how bad the developed model is and avoids overfitting to achieve a minimum value of loss and obtain optimal bias and weight values. The loss function equation [37] is shown in Equation (2).

(2) Lpi,ti=1Ncls∑iLclspi,pi∗+α1Nreg ∑ipi∗Lregti,ti∗ with j=1,⋯,m,

where *L* is the loss function, *L*_*c* *l* *s*_ and *L*_*r* *e* *g*_ are the classification loss and localization losses, *N*_*c* *l* *s*_ and *N*_*r* *e* *g*_ are the numbers of matched boxes for classification and localization, and *α* is the scaling factor for localization loss.

#### 2.2.4. Testing Model and Data Analysis

In this study, we used an input dimension of 224 × 224 × 3 in accordance with the standard ResNet‐50 architecture. The main hyperparameter configurations applied in the model training process include a batch size of 100, as well as variations in the training steps or epoch: 1000, 3000, and 5000. The model was tested by entering data input as testing data for each testing session with a cross‐validation technique. We used evaluation parameters, namely accuracy, precision, recall, and F1‐score values, to analyze and test the performance of the data obtained. These parameters are obtained from the confusion matrix component by knowing true positives (TPs), true negatives (TNs), false positives (FPs), and false negatives (FNs). The equations for the evaluation parameters [38, 39] are shown in Equations (3–6).

(3) Accuracy=TP+TNTP+FP+TN+FN.

(4) Precision=TPTP+FP

(5) Recall=TPTP+FN.

(6) F1−score=2×Precision∗RecallPrecision+Recall.

## 3. Results and Discussion

We used three types of configuration variations in the training: 1000, 3000, and 5000 training steps. The augmented data were trained on the model with the three configurations. The loss function values per step provided by the TensorFlow Object Detection API were extracted from the training process and entered into the TensorBoard visualization tool. There were three loss functions that described the rate of error reduction for the model to detect object classification, object localization, and the average of both. The learning rate graph illustrates the significance of the learning process of the model.

The computational time required for training variations was 15 min for 1000 training steps, 30 min for variations of 3000 training steps, and 40 min for variations of 5000 training steps. Figure 6a shows that the loss function of the classifier layer during model training for 1000 training steps reduced from 0.14 to 0.08, for 3000 training steps to 0.04, and for variations of 5000 training steps to 0.02. The error value itself is the difference between the probability value of the model classification of an object in the image and the object label in the ground truth evaluation data. Figure 6b shows that the loss function of the RPN during model training for 1000 training steps, with the error value reduced from between 0.052 to 0.017; for 3000 training steps to 0.005; and for 5000 training steps to 0.001. Figure 6c shows that the total loss from the variation of 1000 training steps reduced the error from between 0.5 to 0.3; for the variation of 3000 training steps by up to 0.1; and for the variation of 5000 training steps to 0.05. It is clear that the more training steps used, the smaller the error value reduction from classification, localization, and an average of both. Figure 6d shows that the learning rate for 1000 training steps is still increasing, starts to experience a stable condition at 3000 training steps, and decreases after going to 5000 training steps.

Figure 6Training results for different training step variations: (a) classification loss, showing a decrease in error as training steps increase, with the lowest error at 5000 steps; (b) localization loss from the region proposal network (RPN), which also decreases with more training steps; (c) total loss, combining classification and localization loss, showing that more training steps lead to better overall performance; and (d) learning rate trend during training, which increases at 1000 steps, becomes stable at 3000 steps, and starts to decrease at 5000 steps.

**Figure:**
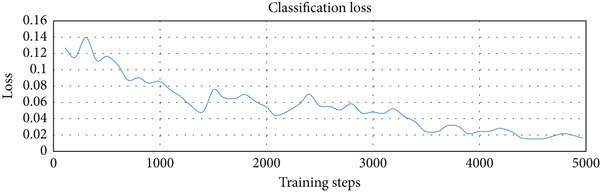
(a)

**Figure:**
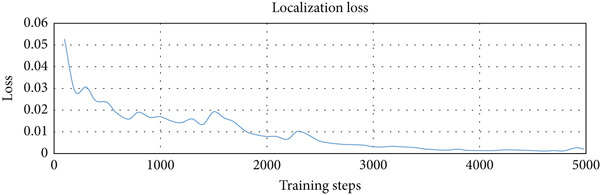
(b)

**Figure:**
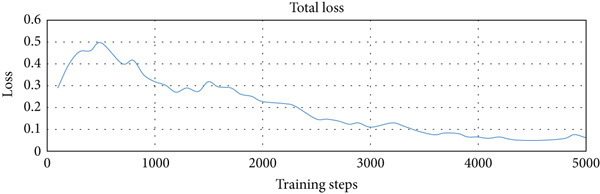
(c)

**Figure:**
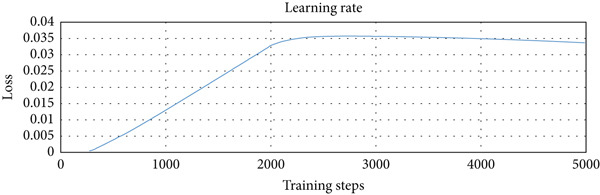
(d)

**Figure 7:**
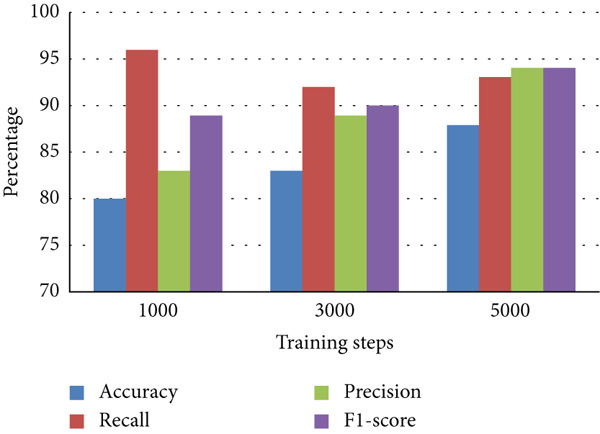
Evaluation metrics for each model configuration in bar chart. This figure compares the performance of the object detection model trained with different training steps (1000, 3000, and 5000 steps) using accuracy, precision, recall, and F1‐score. The results show how increasing training steps affect overall model performance in detecting TB bacteria.

Table 1 is a comparison of the parameter values for the three variations of the training steps, shown as a bar chart in Figure 7. It is evident that the Faster R‐CNN algorithm can recognize *M. tuberculosis* objects well for all variations, with the values of accuracy, precision, and F1‐score increasing with the addition of more training steps. The increase in the accuracy of the model illustrates an increase in the model′s ability to correctly distinguish bacterial objects from ground truth. The increase in the F1‐score itself signifies an increase in the model′s ability to detect the correct object and distinguish that object from nonobject classes. The recall value tends to decrease along with the addition of more training steps. The recall value describes the model′s ability to recognize the potential for the object to be high at the beginning of the training due to the lack of a model describing the type of object that needs to be identified. The increase in the values of precision and accuracy along with a decrease in the recall value illustrates that the model recognition of specific objects has improved. In this study, using the ResNet architecture coupled with image enhancement algorithms such as hue variation, saturation stretching, brightness stretching, and exposure stretching results in a fast computation time and good accuracy. Table 2 shows a comparison of our findings with previously published findings. Figure 8 shows the images of the sputum smear with bounding boxes and the percentages compared with the ground truth data.

**Table 1:** Comparison of evaluation metrics (true positive, true negative, false positive, false negative, accuracy, precision, recall, and F1‐score) for three model configurations trained at 1000, 3000, and 5000 steps. The table illustrates how increasing the number of training steps affects model performance in detecting TB bacteria.

**Parameters**	**1000 training steps**	**3000 training steps**	**5000 training steps**
True positive	70	66	66
True negative	—	—	—
False positive	14	8	4
False negative	3	6	5
Accuracy	80%	83%	88%
Recall	96%	92%	93%
Precision	83%	89%	94%
F1‐score	89%	90%	94%

**Table 2:** Comparison of our proposed method with previous research in terms of dataset type, methodology, and performance results. This comparison highlights the strengths and differences of various approaches used for TB bacteria detection from sputum smear images.

**Authors**	**Dataset**	**Method**	**Highest result**
Panicker et al. (2018) [9]	22 sputum smear microscopic images prepared at the (Instituto Nacional de Pesquisas da Amazonia) lab, Manaus, Brazil	Proposed CNN architecture with augmentation techniques (adding vertical and horizontal reflections, rotation of 180 degrees of images in the training set and extend the number of training samples)	Precision = 0.784
			Recall = 0.971
			F1 − score = 0.867
El‐Melegy et al. (2019) [24]	500 Images of sputum from Ziehl–Neelsen sputum smear microscopy image dataBase (ZNSM‐iDB), Indira Gandhi Medical College, Shimla	Faster R‐CNN with VGG‐16 architecture with data augmentation (random image rotation, mirroring, and random translation)	Precision = 0.826
			Recall = 0.983
			F1 − score = 0.897
Rachmad et al. (2020) [40]	50 images of sputum from ZNSM‐iDB, Indira Gandhi Medical College, Shimla	Adaptive boosting and expert system (random forest) classifier	Accuracy = 0.84
			Precision = 0.52
			Recall = 0.85
			F1 − score = 0.64
El‐Melegy et al. (2020) [7]	500 images of sputum from ZNSM‐iDB, Indira Gandhi Medical College, Shimla	Faster R‐CNN (VGG‐16) followed by second stage classifier CNN with data augmentation (rotation, reflection, and translation)	Precision = 0.851
			Recall = 0.984
			F − score = 0.912
Rachmad et al. (2021) [26]	50 images of sputum from ZNSM‐iDB, Indira Gandhi Medical College, Shimla	Adaptive boosting with decision tree classifier	Accuracy = 0.817
			Precision = 0.524
			Recall = 0.814
			F1 − score = 0.634
Muyama et al. (2021) [25]	148 Ziehl‐Neelsen stained sputum smear microscopic images from The Artificial Intelligence Research Laboratory situated at the College of Computing and Information Sciences and online database from ZNSM‐iDB	CNN with VGGNet and GoogLeNet Inception v3 architecture with and without data augmentation (rotation or reflection of the image, zooming in and out, shifting, distortion) and with fine‐tuning	VGG Net
			Accuracy = 0.80
			Precision = 0.795
			Recall = 0.867
			Inception v3
			Accuracy = 0.867
			Precision = 0.789
			Recall = 1.00
Chen et al. (2024) [41]	1265 sputum images and 3734 bounding boxes of M. Tuberculosis	YOLOv8s‐based model with multiscale feature fusion (MSFF) and enhanced attention (ECCSA).	Average Precision = 85.7*%*
Proposed Method	600 Sputum Smear Images with multivariants Ziehl–Neelsen stain from Universitas Airlangga, Indonesia	CNN with ResNet‐50 architecture with data augmentation (random rotation, random flipping, and color processing like hue variation, saturation stretching, brightness stretching, and exposure stretching)	Accuracy = 0.88
			Precision = 0.94
			Recall = 0.96
			F1 − score = 0.94

Figure 8Visualization of detection results (a) percentage of bounding boxes predicted by the model across different categories or regions in the image and (b) comparison between predicted bounding boxes (in red) and ground truth annotations (in black), illustrating the model′s detection accuracy and spatial alignment with actual TB bacteria locations.

**Figure:**
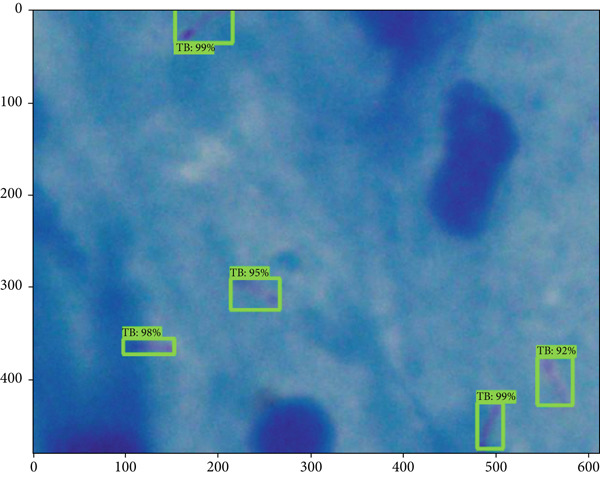
(a)

**Figure:**
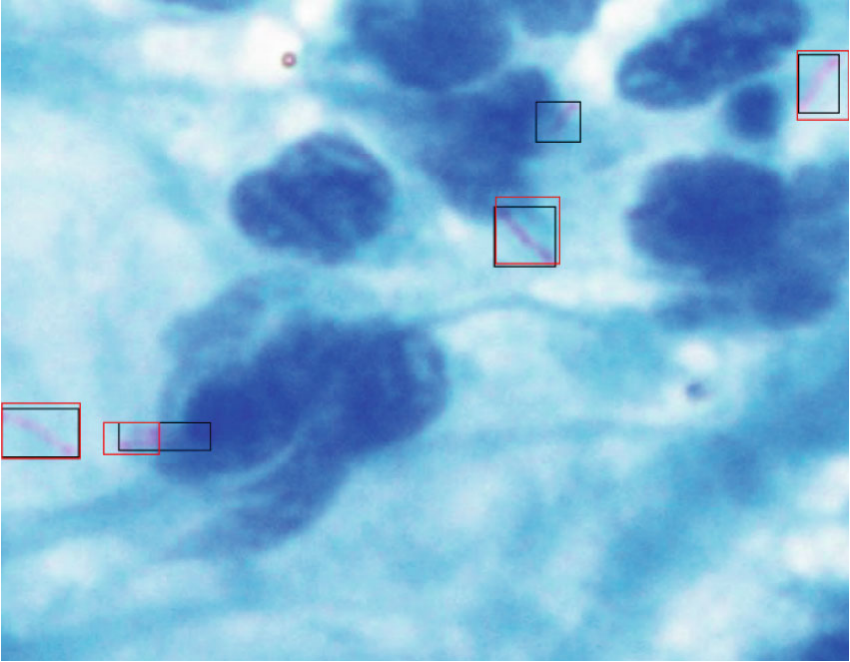
(b)

## 4. Conclusion

We developed a detection system for *M. tuberculosis* bacteria using the Faster R‐CNN algorithm with the ResNet‐50 architecture. The data used consisted of 600 images of sputum smears with Ziehl–Neelsen staining with various staining conditions (thick, medium, and thin) obtained from a conventional microscope. The test results showed an accuracy of 88%, precision of 94%, recall of 96%, and F1‐score of 94%. These results can be influenced by variations in the number of training steps. We used 1000, 3000, and 5000 training steps. The model used is able to distinguish which objects are TB bacteria and which are not. This research can be used as a basis for the automated detection of TB bacteria.

## Conflicts of Interest

The authors declare no conflicts of interest.

## Funding

No funding was received for this manuscript.

## Data Availability

The data can be accessed in https://drive.google.com/drive/folders/1-6Apz5KYbH2pDtMo80bEKEUwVxKu974L.
